# Antinociceptive, anti-inflammatory, and anti-dysmenorrheal activities of aerial parts of *Cannabis sativa* L. from the sub-middle region of the Vale do São Francisco

**DOI:** 10.3389/fphar.2025.1677987

**Published:** 2025-09-29

**Authors:** Pedro Modesto Nascimento Menezes, João Lázaro de Oliveira Rocha, Murilo Soares Silva, Juliane Maria dos Santos Silva, Tarcísio Cícero de Lima Araújo, Deborah Lays Silva Deus, Pedro Jose Rolim-Neto, Luana Fernandes Matos, Ana Beatriz Rodrigues Massaranduba, Fabrício Souza Silva, Larissa Araújo Rolim

**Affiliations:** ^1^ Laboratório de Farmacologia Experimental (LAFEX), Universidade Federal do Vale do São Francisco, UNIVASF, Petrolina, Pernambuco, Brazil; ^2^ Central Analítica de Fármacos, Medicamentos e Alimentos (CAFMA), Universidade Federal do Vale do São Francisco, UNIVASF, Petrolina, Pernambuco, Brazil; ^3^ Grupo de Pesquisa e Extensão Tecnológica em Cannabis Medicinal (GPETCAM), Universidade Federal do Vale do São Francisco, UNIVASF, Petrolina, Pernambuco, Brazil; ^4^ Universidade Federal de Pernambuco, UFPE, Recife, Pernambuco, Brazil; ^5^ Núcleo de Estudos em Plantas Medicinais (NEPLAME), Universidade Federal do Vale do São Francisco, UNIVASF, Petrolina, Pernambuco, Brazil; ^6^ Pós-Graduação em Ciências da Saúde e Biológicas (PGCSB) UNIVASF, Petrolina, Pernambuco, Brazil

**Keywords:** chemical analysis, entourage effect, ethnopharmacology, marijuana, preclinical study

## Abstract

**Introduction:**

*Cannabis sativa* L. has been used for thousands of years to treat intestinal and uterine diseases and as an anti-inflammatory, analgesic, and antiepileptic, among others. This study aimed to conduct preclinical studies based on the ethnopharmacological properties of *C. sativa*.

**Methods:**

For this purpose, the police and health authorities provided the raw plant material, and a crude ethanolic extract of the aerial parts of *C. sativa* (APCs) was produced, which was subsequently chemically analyzed using combined chromatographic and spectrometric methods. Subsequently, APCs were administered to Swiss mice and Wistar rats for evaluation using the open field test, acetic acid-induced abdominal contraction model, hot plate test, formalin test, carrageenan-induced paw edema, *Saccharomyces cerevisiae*-induced fever, and primary dysmenorrhea models.

**Results:**

Chemical analysis suggests the presence of classic cannabinoids, such as cannabidiol, tetrahydrocannabinol, and cannabigerol, as well as flavonoids and alkaloids. The doses used in the open field test were 1, 3, 10, 30, and 100 mg/kg (gavage, po), with the last two doses responsible for reducing mobility and inducing hypothermia in the animals. In subsequent pharmacological protocols, the doses used were 1, 3, and 10 mg/kg (gavage, po). In the abdominal contraction model, the number of writhing events was reduced by APCs at a dose of 10 mg/kg [median 0.5 (Q25 = 0; Q75 = 5.75, p < 0.05)]. In the hot plate test, the doses of 1, 3, and 10 mg/kg increased the latency time to 17.67 ± 1.33, 18.50 ± 1.31, and 17.33 ± 1.69 s (p < 0.05), respectively. In the formalin test, the effect was restricted to the first phase, with values of 42.33 ± 7.588, 45.50 ± 6.657, and 39.50 ± 7.869 s (p < 0.05) in paw-licking time. In paw edema, the doses of 1 and 3 mg/kg were more constant, restricting the volume to 0.168 ± 0.004 and 0.150 ± 0.004 mL (p < 0.05), respectively. In dysmenorrhea, the doses of 3 and 10 mg/kg reduced abdominal contractions [0 (Q25 = 0; Q75 = 3.0) and 1.0 (Q25 = 0; Q75 = 3.0)].

**Conclusion:**

APCs at the tested doses did not promote an antipyretic effect. These data indicate that APCs have antinociceptive, anti-inflammatory, and anti-dysmenorrheal effects in animal models.

## Highlights

1. Classic cannabinoids, flavonoids, and alkaloids are found in APCs.
2. APCs had a hypothermic effect and reduced the mobility of mice.
3. APCs showed antinociceptive, anti-inflammatory, and anti-dysmenorrheic effects.
4. APCs had no antipyretic effect at non-hypothermic doses.

## 1 Introduction

*Cannabis sativa* L. is a plant from the family Cannabaceae and one of the oldest to be domesticated in the world, with its use dating back to approximately 12,000 years in the Central Asian region (McPartland, 2018; Duchateau et al., 2025). The number of identified chemical substances continues to expand as research progresses on their medicinal use, with the identification of more than 500 compounds, including around 125 classified as phytocannabinoids (Salehi et al., 2022; Maccarrone, 2022; Fordjour et al., 2023).

*C. sativa* has a variety of indications in traditional medicine, in the most diverse forms of use (tea, smoke, vapor, etc.), and is used as a wound healing agent, analgesic, anticonvulsant, hypnotic, tranquilizer, anesthetic, anti-inflammatory, antibiotic, antiparasitic, antispasmodic, digestive, appetite stimulant, diuretic, aphrodisiac, antitussive, and expectorant (Zuardi, 2006; Pisanti and Bifulco, 2019; Maurya et al., 2021).

Many studies have already been conducted on the plant, but the vast majority of them are mainly aimed at using the classic cannabinoids obtained, Δ^9^-tetrahydrocannabinol (THC) and cannabidiol (CBD), as they are the first substances identified and are responsible for their psychoactive and medicinal effects (Gaoni and Mechoulam, 1964; Lucas et al., 2018; Fordjour et al., 2023). However, there are important considerations regarding these two compounds, as well as the other chemical substances found in the plant, which can lead to a synergistic effect between these components and potentiate the pharmacological effects already observed with classic cannabinoids (Karniol and Carlini, 1973; Koltai and Namdar, 2020).

Terpenes and other compounds produced by plants can interact synergistically, modulating or enhancing the pharmacological effects of phytocannabinoids on their various targets, such as cannabinoid receptors, transient potential receptors, and peroxisomal receptors (Russo, 2011; Chacon et al., 2022).

Our research group has studied *C. sativa*, mainly the roots, and identified some chemical compounds such as cannabisativine, anhydrocannabisativine, *p*-coumaroyltyramine, and feruloyltyramine, which have pharmacological effects, such as antinociceptive, anti-inflammatory, antitussive, expectorant, antiasthmatic, and anti-dysmenorrheic effects, with the possibility of low toxicity (Lima et al., 2021; Menezes et al., 2021; Menezes et al., 2022; Araújo et al., 2024).

Thus, considering that the extract of the aerial parts has chemical compounds from diverse classes, such as terpenes, flavonoids, and classic cannabinoids (CBD and THC), the chemical-pharmacological investigation of *C. sativa* L. from the Vale do São Francisco region has great pharmaceutical relevance. Furthermore, studies on the aerial parts of *C. sativa* for non-psychoactive effects are rarely found in the literature, except for the use of classic cannabinoids present in the plant. Thus, the objective of our study was to observe the synergistic pharmacological effects of the chemical compounds of *Cannabis* (entourage effect), without inducing behavioral changes in animals, for the treatment of pain, inflammation, and dysmenorrhea, among others, mitigating the effects on the central nervous system (CNS) that are classic with the medicinal use of *C. sativa*.

## 2 Materials and methods

### 2.1 Plant materials

The aerial parts of *C. sativa* were collected and donated by the Brazilian Federal Police Department. The plants were the result of seizures in illegal plantations in a city in the state of Bahia, Brazil (latitude: 08 ° 59′ 25″ S and longitude: 39 ° 54′ 34″ W, in February 2018), and the studies were authorized by the Brazilian Federal Court, National Health Surveillance Agency, and National System for the Management of Genetic Heritage and Associated Traditional Knowledge. The sample was identified by the Herbário Vale do São Francisco (HVASF) by comparison with the deposit under the code 23.331 of the Universidade Federal do Vale do São Francisco (UNIVASF).

With the plant available, the aerial parts were immediately separated from the rest of the plant, treated, and dried in a circulating air oven at an average temperature of 40  °C for 72 h. After this process, the plant material was pulverized in a mill to obtain a powder, which was stored in an inert container at 15  °C ± 1  °C.

### 2.2 Preparation of the *C. sativa* crude ethanolic extract (APCs)

To prepare the ethanolic extract of the aerial parts of *C. sativa* (APCs), previously ground *C. sativa* plant material (0.3 kg) was placed in a container containing 1 L of absolute ethanol (99.5%). This process was carried out until the extractive process was exhausted, as indicated by the solvent retaining its original color (Slatkin et al., 1971).

The solvent was evaporated using a rotary evaporator at 40 °C to obtain a crude ethanolic extract of *C. sativa*.

### 2.3 APCs chemical analysis

Liquid Chromatography-Mass Spectrometry (LC-MS) analyses were performed using a Prominence^®^ Shimadzu^®^ system equipped with a Diode Array (DAD) coupled to a Bruker^®^ mass spectrometer equipped with an electrospray ionization (ESI) source. A reversed-phase column was used for chromatographic separation at 30 °C. Chromatography was performed in gradient mode using the solvent mixture used in the chromatographic analyses as the eluent. The flow was maintained at 1.4 mL·min^−1^ with part of the eluent directed to the mass spectrometer via a splitter at a flow rate of 150 µL·min^−1^. The chromatogram was monitored at wavelengths 200 and 800 nm. The mass spectrometer was operated under the following conditions: 3 kV electrospray probe and 25 V cone; N2 gas for nebulization at flow rates of 345 L·h^−1^ and 27 L·h^−1^, respectively, at 180 °C. The collision cell was filled with N2 at 7 psi, and the collision energy was varied from 15 to 40 eV for the MS/MS experiments.

The chromatographic peaks obtained in the LC-DAD-MS/MS analyses were identified by comparative evaluation with the molecules described in the literature using the following parameters: pure ultraviolet (UV) spectrum, accurate mass of the ions ([M + H]+, [M + Na]+, and [M + K]+) at high resolution, and product ion spectra generated in MS/MS experiments from collision-induced dissociation. Substances with errors not exceeding 15 ppm were considered to be detected. A molecular network was created using the protocol available on the Global Natural Products Social Molecular Networking (GNPS) website. The data were filtered by removing all MS/MS fragment ions within ±17 Da of the m/z ratio of the precursor ion. The MS/MS spectra were filtered by selecting the six most intense peaks in the ±50 Da window across the entire spectrum. The tolerance for the precursor ion was set at 2.0 Da and for the fragment ion at 0.5 Da.

### 2.4 Animals

Male Swiss mice (*Mus musculus*) were used to perform the experiments. The mice used were between 4 and 6 weeks old and weighed between 30–40 g. Young Wistar rats (*Rattus norvegicus*) aged 28–31 days and weighing 75–90 g were used in the fever protocol. All animals were obtained from the Central Animal Facility of Universidade Federal do Vale do São Francisco (UNIVASF). The animals were kept randomly in polypropylene boxes with grilled lids and access to water and food (pelletized commercial feed for mice and rats, Presence^®^, Brazil) at a controlled temperature (22  °C ± 2 °C) and 12-h light/dark cycles. The animals were fasted for 1 h before the test.

The experimental protocols were submitted to UNIVASF’s Ethics Committee on the Use of Animals (CEUA) for authorization of the experiments, which was approved on 10/10/2022 under the number 0003/310822. All experimental protocols were carried out in accordance with current legislation under the guidance of the CEUA-UNIVASF, the National Council for the Control of Animal Experimentation (CONCEA), and Animal Research: Reporting of *in vivo* Experiments 2.0 (ARRIVE 2.0) (Sert et al., 2020).

### 2.5 Open field test (OFT)

The OFT is an experimental protocol that aims to assess the behavior of animals after drug administration. Mice (n = 5/group) were weighed for the administration of APCs, orally (po) with a gavage needle, after analysis of the study by Ewing et al. (2019), at doses of 1, 3, 10, 30, and 100 mg/kg (Ewing et al., 2019). One group of animals received distilled water with 3% cremophor as a vehicle for administering the extract at 10 mL/kg body weight. The basal body temperature ( °C) of the animals was assessed. After 30 min of treatment, the mice were placed in the center of the arena to observe for 5 minutes how far the animal walked across the open field, how far it walked through the center of the apparatus or near the edges (Duarte-Filho et al., 2022), as well as post-treatment temperature analysis (Bilbrey et al., 2022). After the 5-min evaluation period for each animal, the apparatus was cleaned with 5% (v/v) ethanol, and the animals were euthanized by cervical dislocation (Carlini and Mendes, 2012).

### 2.6 Abdominal writhing induced by acetic acid

Abdominal contractions are a behavior generated by the action of acetic acid in the mobilization of endogenous nociceptive and inflammatory substances in the peritoneal region. Mice (n = 6/group) were weighed and administered distilled water with 3% Cremophor (vehicle) at 10 mL/kg and APCs at doses of 1, 3, or 10 mg/kg (po) (Ewing et al., 2019). Another 2 groups were positive controls, in which the animals were treated with indomethacin (20 mg/kg) or morphine (10 mg/kg, ip). Thirty minutes after treatment, the mice were administered 0.9% (v/v) acetic acid at a dose of 10 mL/kg (ip). Immediately after acetic acid injection, the mice were placed in an apparatus with mirrored walls, and the number of contortions was counted for 20 min. The contortions were characterized by perceptible contractions of the abdomen followed by trunk rotation and hind limb extension, generated by reflex stimuli produced by irritation after the injection of acetic acid (Whittle, 1964; Yin et al., 2016).

### 2.7 Hot plate test

The hot plate is a device that allows thermal stimulation of animals’ nerve endings, generating nociceptive behavior. The mice (n = 6/group) were pretested on a hot plate (Insight^®^, Brazil) at 55  °C ± 0.5 °C. Animals that reacted to the thermal stimulus after only 10 s of exposure were excluded from the test to ensure sample homogeneity. Lifting and/or licking the paws was considered a response to thermal stimulus. After selection, the animals were weighed and administered distilled water with 3% Cremophor (vehicle) at 10 mL/kg, APCs at doses of 1, 3, and 10 mg/kg (po), or morphine at 10 mg/kg (ip). After 30 min, the animals were placed individually on the previously heated plate and observed at 30, 60, 90, and 120 min after treatments application. The time taken for the animal to exhibit a painful reaction was measured using a stopwatch, and each animal was exposed to the hot plate for a maximum of 20 s to avoid tissue damage (Franzotti et al., 2000; Deng et al., 2021).

### 2.8 Formalin-induced nociception

In the formalin test, animals are exposed to intraplantar administration of a mixture of formaldehyde and saline to generate animal behavior, such as licking, biting, or paw shaking, in two distinct phases, which indicates nociception stimulated by the CNS and inflammatory mediators, respectively. The mice (n = 6/group) were weighed and administered distilled water with 3% cremophor (vehicle) at 10 mL/kg and APCs at 1, 3, or 10 mg/kg (po). Another 2 groups made up as positive controls, in which the animals were treated with nimesulide (100 mg/kg, po) or morphine (10 mg/kg, ip). One hour after treatment administration, the animals underwent intraplantar application of 2.5% (v/v) formalin solution. A volume of 20 µL of the solution was injected into the right hind paw of each animal, which was placed in a glass chamber with mirrored walls, and the time taken to lick, shake, or bite the injured paw was recorded as a sign of nociception. The time was assessed in two observation phases (0–5 min and 20–30 min) (Hunskaar and Hole, 1987; Fischer et al., 2014; Liu et al., 2021).

### 2.9 Carrageenan-induced paw edema

In paw edema, carrageenan is used as an inflammatory agent, inducing the migration of inflammatory mediators and variation in the volume of the paws of the animals, which will be evaluated in the plethysmometer. The mice (n = 6/group) were weighed and administered distilled water with 3% cremophor (vehicle) at 10 mL/kg, APCs at doses of 1, 3, or 10 mg/kg (po), or nimesulide 100 mg/kg (po). Paw edema was induced in all animals by subcutaneous administration of 100 µL of 1% (w/v) carrageenan suspension on the planar surface of the left hind paw of each animal. In one group, saline solution was administered to observe the appearance of edema without a phlogistic agent. The progression of edema in the animal paws was assessed using a digital plethysmometer with a solution prepared with 0.05% (w/v) NaCl and Tryton. Paw volumes were measured at 30, 90, 150, and 210 min after carrageenan administration (Costa et al., 2004; Alabi et al., 2024).

### 2.10 Antipyretic activity evaluation

Yeast-induced antipyretic activity is a method in which there is a systemic stimulus for the mobilization of the immune system, inducing the release of inflammatory mediators, mainly in the CNS. Young rats (n = 5/group) had their basal rectal temperatures measured for three consecutive days before the start of the test (insertion of a 2.5 cm thermometer with the aid of liquid vaseline). On the fourth day, fever was induced by intraperitoneal injection of a suspension of *Saccharomyces cerevisiae* at a dose of 135 mg/kg in a sterile saline solution. In one experimental group, the animals received only saline solution to observe basal temperature. Four hours after the yeast injection, the animals were administered distilled water with cremophor 3% (vehicle) at 10 mL/kg, APCs (1, 3, or 10 mg/kg, po), or dipyrone (120 mg/kg, ip). The rectal temperature of all animals was measured every hour for 8 h (Tomazetti et al., 2005; Abotsi et al., 2017).

### 2.11 Anti-dysmenorrheal evaluation

In the primary dysmenorrhea protocol, abdominal contortion in animals is observed after hormonal treatment and uterine contractions, thus providing a nociceptive stimulus. Virgin female mice (n = 7/group) were used to induce experimental primary dysmenorrhea (Yang et al., 2015; Araújo et al., 2024). For the experimental protocol, estradiol benzoate 1 mg/kg/day (ip) was administered for three consecutive days in the groups that would receive subsequent treatment, with one group (n = 6) not receiving estradiol, but saline (10 mL/kg). On the 4th day, a 1-h fast and the treatments were administered according to each experimental group, receiving distilled water with cremophor 3% (vehicle) at 10 mL/kg, APCs at doses of 1, 3, or 10 mg/kg, or mefenamic acid 90 mg/kg (po). All treatments were administered only once. After 30 min of administration in the respective groups, 0.4 UI of oxytocin (ip) was injected into each mouse. Subsequently, the animals were placed in a mirrored glass chamber to record and observe abdominal contortions (contraction of the abdominal wall, pelvic rotation, and stretching of the hind legs) for 30 min. At the end of the experiment, all animals were euthanized.

### 2.12 Statistical analysis

All results are expressed as mean ± standard error of the mean (SEM) or median with interquartile range (25% and 75% - Q25–Q75). Statistically significant differences between the experimental and control groups were calculated using Student’s t-test or one-way or two-way analysis of variance (ANOVA) followed by Dunnett’s post-test, depending on the experimental protocol, for parametric data. However, for non-parametric data, statistical analysis was carried out using Kruskal-Wallis, followed by Dunn’s post-test or the Mann-Whitney test, when necessary.

Sample size estimation was performed using G*Power based on data from previous studies employing comparable experimental protocols. The parameters were defined as follows: one-way analysis of variance (ANOVA) for hypothesis testing, an average standard deviation of 35% of the experimental response, a significance level of 0.05 (Type I error), and expected differences between the control and experimental groups ranging from 10% to 80%. Under these assumptions, the required sample size was n = 7 per group, yielding a statistical power of 0.96.

Statistical significance was set at *p* < 0.05. GraphPad Prism^®^ software version 8.0 for Windows was used to plot the data and performed statistical analysis.

## 3 Results

### 3.1 Chemical analysis of APCs by LC-MS (ESI-IT)

The fragmentation data (Table 1) detected in comparison with the literature suggests the presence of the classic cannabinoids: CBD, THC, cannabichromene (CBC), cannabigerol (CBG), cannabinol (CBN), cannabicitran (CBT), cannabidivarin (CBDV), tetrahydrocannabivarin (THCV), some derivatives such as cannabinol-d3 (CBNd3), cannabidivarin acid (CBDVA), and cannabidiolic acid (CBDA), among other substances that are products of secondary metabolism of biological importance, the alkaloid cannabisativin (CBS), and some compounds of the flavonoid class: Vitexin-2″-O-ramnoside, Isovitexin, 2′-O-Glucosylvitexin, Luteolin-7-C-rutinoside, Luteolin-8-C-glucoside, Chrysoeriol-8-C-glucoside and Cannaflavin.

**TABLE 1:** Compounds suggested in APCs by LC-MS (ESI-IT).

Compound	Rt (min)	+MS *m/z*	+MS^2^ *m/z*
CBD	52.7	• 315.26	• **193.10** (Sempio et al., 2021; Takashina et al., 2022) • **259.16** (Sempio et al., 2021) • **107.11** (Barhdadi et al., 2023)
THC	52.8	• 315.23	• **135.16** (Ballotari et al., 2025) • **193.13** (Takashina et al., 2022; Manca et al., 2022) • **259.16** (Manca et al., 2022) • 93.12 • 297.25
CBC	52.8	• 315.24	• **259.12** (Galant et al., 2022) • **193.12** (Galant et al., 2022; Pavlovic et al., 2019) • 109.08 • 297.15
CBT	52.8	• 315.24	• **135.10** (Barhdadi et al., 2023) • **193.12** (Barhdadi et al., 2023; Pavlovic et al., 2019) • **259.12** (Barhdadi et al., 2023)
CBG	46.8	• 317.28	• **193.06** (Sempio et al., 2021; Takashina et al., 2022; Galant et al., 2022; Nemeškalová et al., 2020) • 261.22
CBDV/THCV	46.9	• 287.20	• **231.11** (Sempio et al., 2021) • **165.05** (Sempio et al., 2021; Barhdadi et al., 2023; Nemeškalová et al., 2020) • 93.12
CBN	70.6	• 311.20	• **223.07** (Sempio et al., 2021; Takashina et al., 2022; Barhdadi et al., 2023; Galant et al., 2022; Nemeškalová et al., 2020) • **293.15** (Sempio et al., 2021; Galant et al., 2022; Nemeškalová et al., 2020)
CBNd3	18.1	• 314.16	• **145.00** (Sempio et al., 2021) • **177.01** (Sempio et al., 2021)
CBS	7.0	• 382.27	• **112.11** (Lima et al., 2021; Araújo et al., 2024) • **198.09** (Lima et al., 2021) • **226.16** (Lima et al., 2021; Araújo et al., 2024) • **264.16** (Lima et al., 2021; Araújo et al., 2024) • **364.32** (Lima et al., 2021; Araújo et al., 2024)
*p*-coumaroyltyramine	17.5	• 284.11	• **147.02** (Pavlovic et al., 2019; Yamamoto et al., 1991; Araújo et al., 2024)
THCVA/CBDVA	48.6	• 331.20	• **313.21** (Galant et al., 2022)
CBDA	34.6	• 359.24	• **341.22** (Galant et al., 2022; Nemeškalová et al., 2020) • **219.09** (Takashina et al., 2022; Galant et al., 2022) • 123.09 • 303.18
Cannaflavin	45.2	• 437.17	• **313.08** (Pavlovic et al., 2019)
Vitexin-2″-O-ramnosideo	12.2	• **579.17** (Wen et al., 2017; Xu et al., 2009)	• **433.10** (Li et al., 2022) • **415.06** (Li et al., 2022) • **313.08** (Li et al., 2022) • **271.07** (Li et al., 2022)
Isovitexin	12.6	• **433.11** (Shu et al., 2025)	• **313.06** (Montevecchi et al., 2019; Chebchoub et al., 2024) • **415.10** (Chebchoub et al., 2024) • 367.11
2′-O-Glucosylvitexin	11.8	• 595.12	• **271.05** (Franke et al., 2006) • **313.06** (Franke et al., 2006) • **433.10** (Franke et al., 2006) • 475.08
Luteolin-7-C-rutinoside	11.3	• **595** (Xu et al., 2009)	• **449.11** (Li et al., 2022) • **431.11** (Li et al., 2022) • **329.09** (Li et al., 2022) • **287** (Li et al., 2022)
Luteolin-8-*C*-glucoside	11.4	• 449.07	• **431.07** (Li et al., 2022) • **329.06** (Li et al., 2022) • 383.07
Chrysoeriol-8-C-glucoside	13.5	• 463.11	• **445.10** (Li et al., 2022) • **343.05** (Li et al., 2022) • **397.08** (Li et al., 2022)

Legend: CBD: cannabidiol; THC: tetrahydrocannabinol; CBC: cannabichromene; CBT: cannabicitran; CBG: cannabigerol; CBDV/THCV: cannabidivarin/tetrahydrocannabivarin; CBN: cannabinol; CBNd3: cannabinol d3; CBS: cannabisativine; THCVA/CBDVA: cannabidivarin acid/tetrahydrocannabivarin acid; CBDA: cannabidiolic acid.

The fragments marked in bold are in accordance with the indicated literature.

**FIGURE 1:**
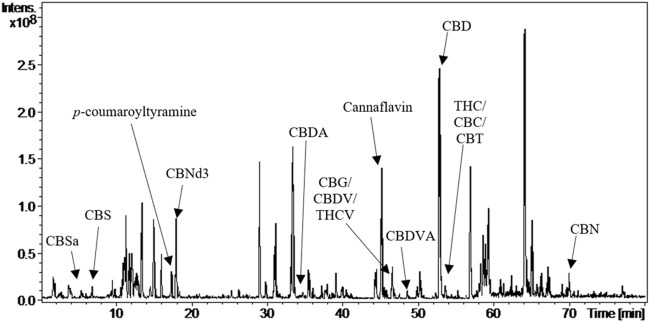
Chromatogram of APCs by LC-MS (ESI-IT) showing the main identified cannabinoids.

Figure 1 shows the chromatogram obtained by LC-MS (ESI-IT) of the APCs and the possible compounds corresponding to each peak.

### 3.2 Evaluation of APCs on rodent behavior in the OFT

The animals were assessed in the open field for their exploratory behavior in the apparatus and their body temperature before and after the administration of APCs at 1, 3, 10, 30, and 100 mg/kg (po).

**FIGURE 2:**
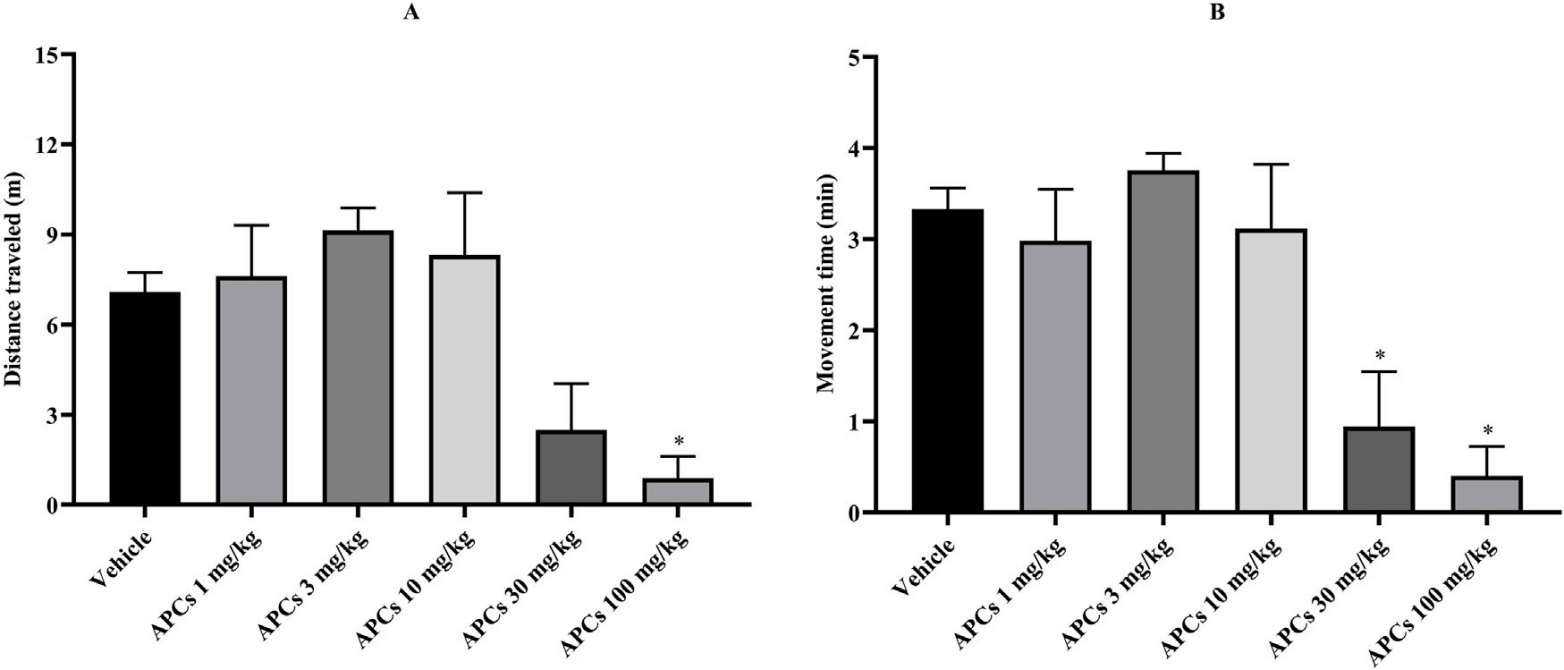
Time and distance covered by the animals in the open field. Legend: Effect of treatment with APCs 1, 3, 10, 30, and 100 mg/kg and vehicle 10 mL/kg on animal behavior. **(A) **the distance traveled by animals in each group was analyzed. **(B)** the time the animal spent moving through the apparatus was analyzed. The results are expressed as the mean ± standard error of the mean (n = 7). *p < 0.05 compared to the negative control group. One-way ANOVA followed by Dunnett’s test.

The behavior was evaluated in terms of the distance traveled (meters) and the time (minutes) that the animal was moving, as shown in Figures 2A,B. In terms of distance, the animals receiving the vehicle traveled 7.086 ± 0.6413 m, while those receiving 100 mg/kg APCs moved for 0.8791 ± 0.7375 m, which showed a significant difference (p < 0.05) between the groups (Figure 2A). The animals treated with APCs at doses of 1, 3, 10, and 30 mg/kg walked 7.613 ± 1.697, 9.139 ± 0.7399, 8.318 ± 2.069, and 2.492 ± 1.546 m, respectively, which was not significantly different from that of the animals that received the vehicle.

In the analysis of movement time, the animals that received the vehicle moved for 3.329 ± 0.2332 min, while those that received APCs at doses of 30 and 100 mg/kg showed a time of 0.9441 ± 0.6003 and 0.4023 ± 0.3228 min, respectively, which represented a significant difference (p < 0.05) between the groups treated with APCs and the vehicle (Figure 2B). On the other hand, the animals that received APCs at doses of 1, 3, and 10 mg/kg had times of 2.981 ± 0.5658, 3.754 ± 0.1880, and 3.113 ± 0.7066 min, respectively, which were not significantly different from the animals that received vehicle.

**FIGURE 3:**
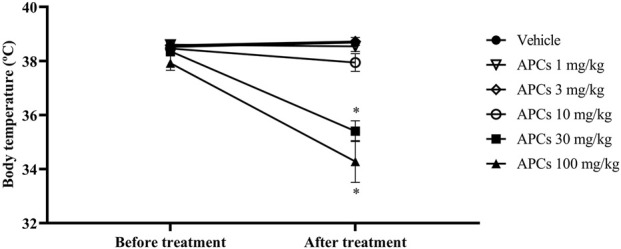
Temperature of the animals after administration of increasing doses of APCs. Legend: Effect of treatment with APCs 1, 3, 10, 30, and 100 mg/kg and vehicle 10 mL/kg on the body temperature of the animals after treatment. The results are expressed as the mean ± standard error of the mean (n = 7). *p < 0.05 compared to the negative control group. One-way ANOVA followed by Dunnett’s test.

Regarding body temperature, there was no significant difference between the animals at the start of the experimental protocol (37.92 °–38.60 °C). However, when the animals were treated with different doses of APCs, there was a significant reduction in temperature (p < 0.05) at 30 mg/kg, where there was a variation from 38.36 °C to 35.40 °C ± 0.3834 °C and for 100 mg/kg, the reduction was from 37.92 °C to 34.28 °C ± 0.7736 °C (Figure 3). The animals that received APCs at doses of 1, 3, and 10 mg/kg had temperatures of 38.57 ± 0.03, 38.65 ± 0.07, and 38.20 °C ± 0.26 °C, respectively, which were not significantly different from those of the animals that received the vehicle.

These data were decisive for defining the dose for subsequent experiments, as there would be no need for behavioral modifications and hypothermia to verify the pharmacological effects involving APCs.

### 3.3 Antinociceptive effect of APCs on abdominal writhing induced by acetic acid

**FIGURE 4:**
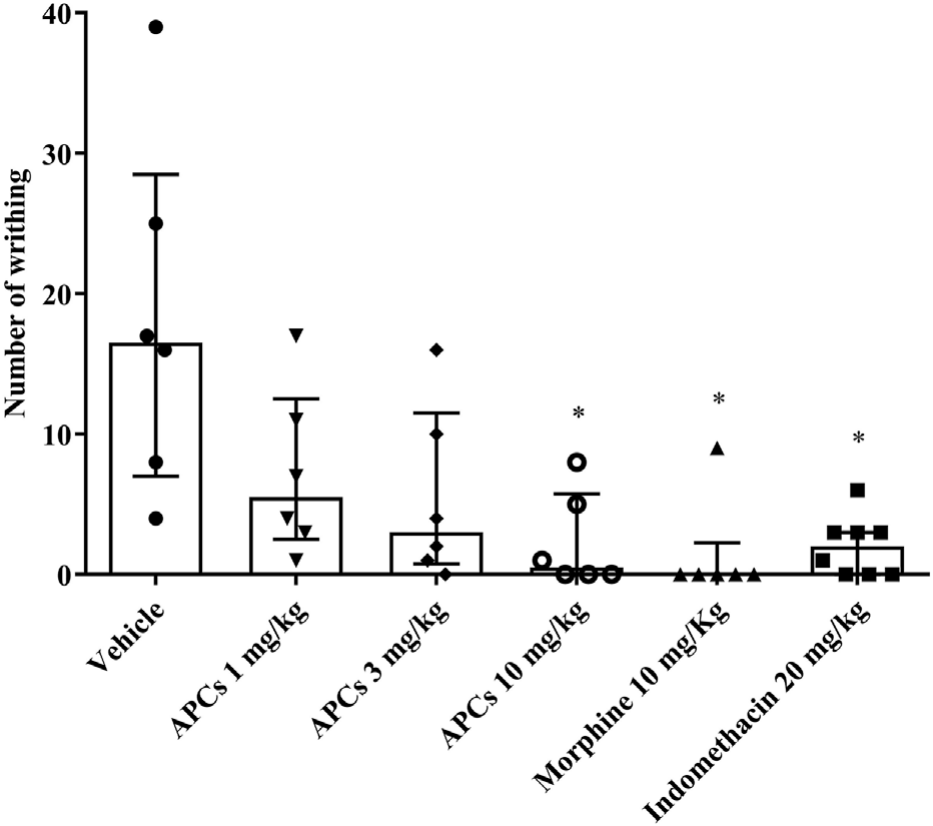
Effect of APCs on acetic acid-induced abdominal contortions. Legend: Effect of treatment with APCs 1, 3, and 10 mg/kg, vehicle 10 mL/kg, and positive controls indomethacin 20 mg/kg and morphine 10 mg/kg on the number of abdominal contortions induced by acetic acid in mice. The results are expressed as median with interquartile range (n = 7). *p < 0.05 compared to the negative control group, by Kruskal-Wallis followed by Dunn’s test.

In the protocol analyzing abdominal contractions induced by acetic acid, a significant reduction (p < 0.05) in this nociceptive behavior as observed when the animals were treated with APCs 10 mg/kg, morphine 10 mg/kg, or indomethacin 20 mg/kg (Figure 4). The animals that received vehicle alone had a median of 16.5 (Q25 = 7; Q75 = 28.5) abdominal contractions, the group treated with APCs 10 mg/kg had 0.5 (Q25 = 0; Q75 = 5.75) abdominal contractions, indomethacin 20 mg/kg had 2.0 (Q25 = 0; Q75 = 3) abdominal contractions, and morphine 10 mg/kg had 0 (Q25 = 0; Q75 = 2.25) abdominal contractions (p < 0.05). The 1 and 3 mg/kg APCs doses resulted in 3.0 (Q25 = 0.75; Q75 = 11.50) and 5.5 (Q25 = 2.5; Q75 = 12.50) abdominal contractions, respectively, with no significant difference compared to the animals that received the vehicle.

### 3.4 Antinociceptive effect of APCs in hot-plate test

**FIGURE 5:**
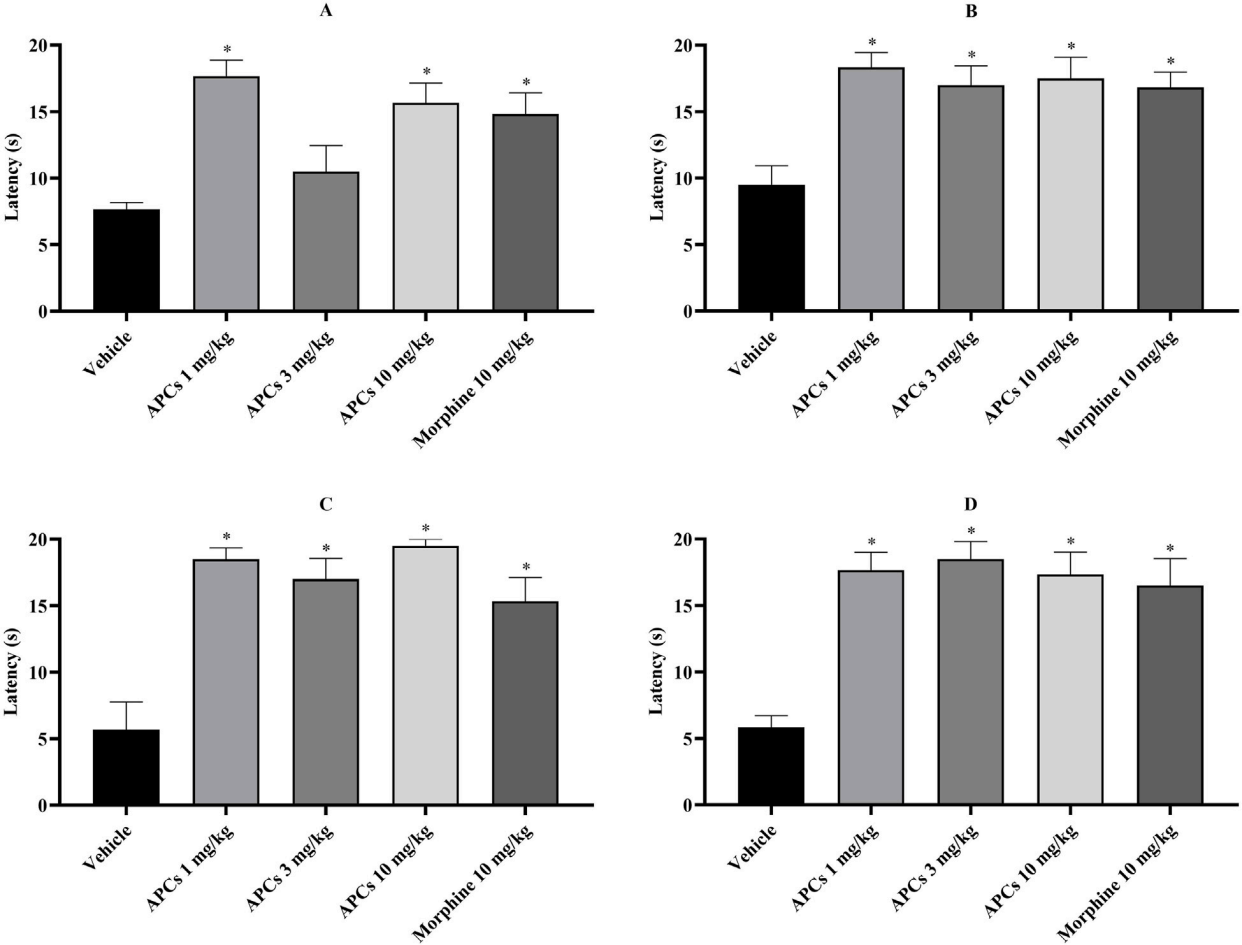
Latency times of the animals on the hot plate. Legend: Effect of treatment with APCs 1, 3, and 10 mg/kg and vehicle 10 mL/kg on the thermal nociceptive behavior of the animals. **(A)** The latency to the stimulus at 30 min was analyzed. **(B)** The latency to the stimulus at 60 min was analyzed. **(C)** The latency to the stimulus at 90 min was analyzed. **(D)** The latency to the stimulus at 120 min was analyzed. The results are expressed as the mean ± standard error of the mean (n = 7). *p < 0.05 compared to the negative control group by one-way ANOVA followed by Dunnett’s test.

In the hot plate test, which shows the time taken for the animal to signal the thermal nociceptive stimulus (latency), APCs at 1, 3, and 10 mg/kg at 30, 60, 90, and 120 min showed a significant difference (p < 0.05) in latency time compared to the animals that received the vehicle, except for APCs 3 mg/kg in the 30-min analysis (Figures 5A–D). Animals treated with morphine (10 mg/kg) also showed significantly longer latency parameters than those that received the vehicle. The numerical data are presented in Table 2.

**TABLE 2:** Latency time data for hot plate test.

Groups	Latency (sec)			
	30 min	60 min	90 min	120 min
Vehicle	7.66 ± 0.49	9.50 ± 1.43	5.67 ± 2.09	5.83 ± 0.87
APCs 1 mg/kg	17.67 ± 1.20*	18.33 ± 1.12*	18.50 ± 0.85*	17.67 ± 1.33*
APCs 3 mg/kg	10.50 ± 1.96	17.00 ± 1.44*	17.00 ± 1.55*	18.50 ± 1.31*
APCs 10 mg/kg	15.67 ± 1.48*	17.50 ± 1.59*	19.50 ± 0.50*	17.33 ± 1.69*
Morphine 10 mg/kg	14.83 ± 1.58*	16.83 ± 1.14*	15.33 ± 1.78*	16.50 ± 2.03*

Legend: *p < 0.05 compared to the negative control group by one-way ANOVA, followed by Dunnett’s test.

### 3.5 Antinociceptive effect of APCs on formalin test

Formalin-induced chemical nociception is divided into two phases, which represent different events, and the animals observed the paw-licking time.

**FIGURE 6:**
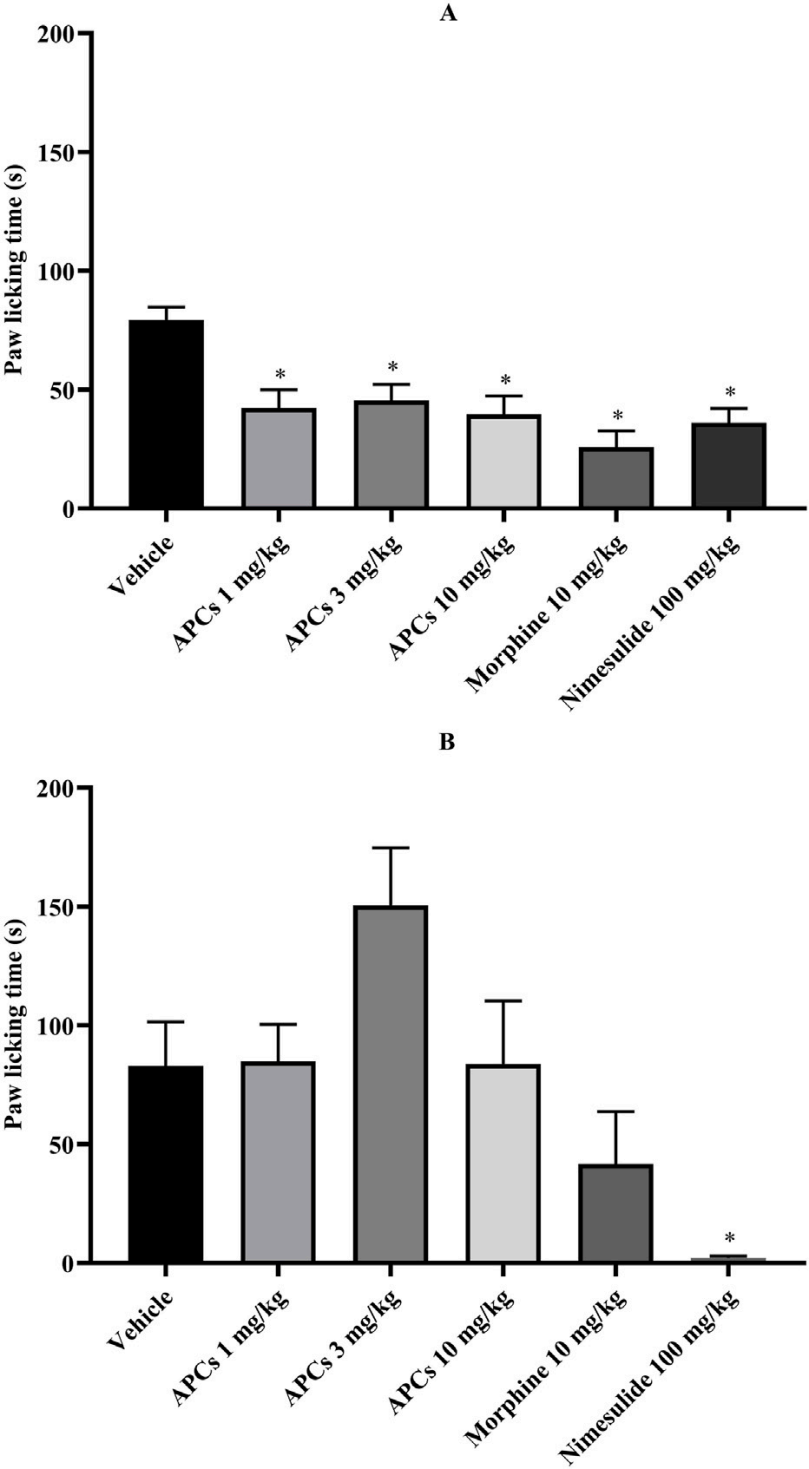
Animal paw-licking time after formalin application. Legend: Effect of treatment with APCs 1, 3, and 10 mg/kg, vehicle 10 mL/kg, morphine 10 mg/kg, and nimesulide 100 mg/kg on chemical nociception behavior. **(A)** The distance walked by animals in each group was analyzed. **(B)** The time the animal spent moving through the apparatus was analyzed. The results are expressed as the mean ± standard error of the mean (n = 7). *p < 0.05 compared to the negative control group. One-way ANOVA followed by Dunnett’s test.

In the first phase, the animals that received the vehicle showed a mean paw licking time of 79.33 ± 5.451 s, which was significantly different (p < 0.05) from that of the animals treated with APCs 1, 3, and 10 mg/kg (42.33 ± 7.588, 45.50 ± 6.657, and 39.50 ± 7.869 s, respectively, Figure 6A). The animals treated with morphine 10 mg/kg and nimesulide 100 mg/kg also showed significant differences compared to the animals that received the vehicle, with values of 25.83 ± 6.680 and 36.00 ± 6.077 s, respectively.

In the second phase, the animals that received vehicle showed the behavior analyzed for 83.00 ± 18.49 s, which was significantly different compared to the animals that received nimesulide 100 mg/kg with values of 2.167 ± 0.7923 s in the paw licking time (Figure 6B). The animals treated with APCs 1, 3, and 10 mg/kg had paw licking times of 84.83 ± 15.59, 150.5 ± 24.27, and 83.67 ± 26.65 s, respectively. The group treated with morphine 10 mg/kg had a licking time of 41.67 ± 22.03 s, which did not represent a significant difference.

### 3.6 Effect of APCs on carrageenan-induced paw edema

**FIGURE 7:**
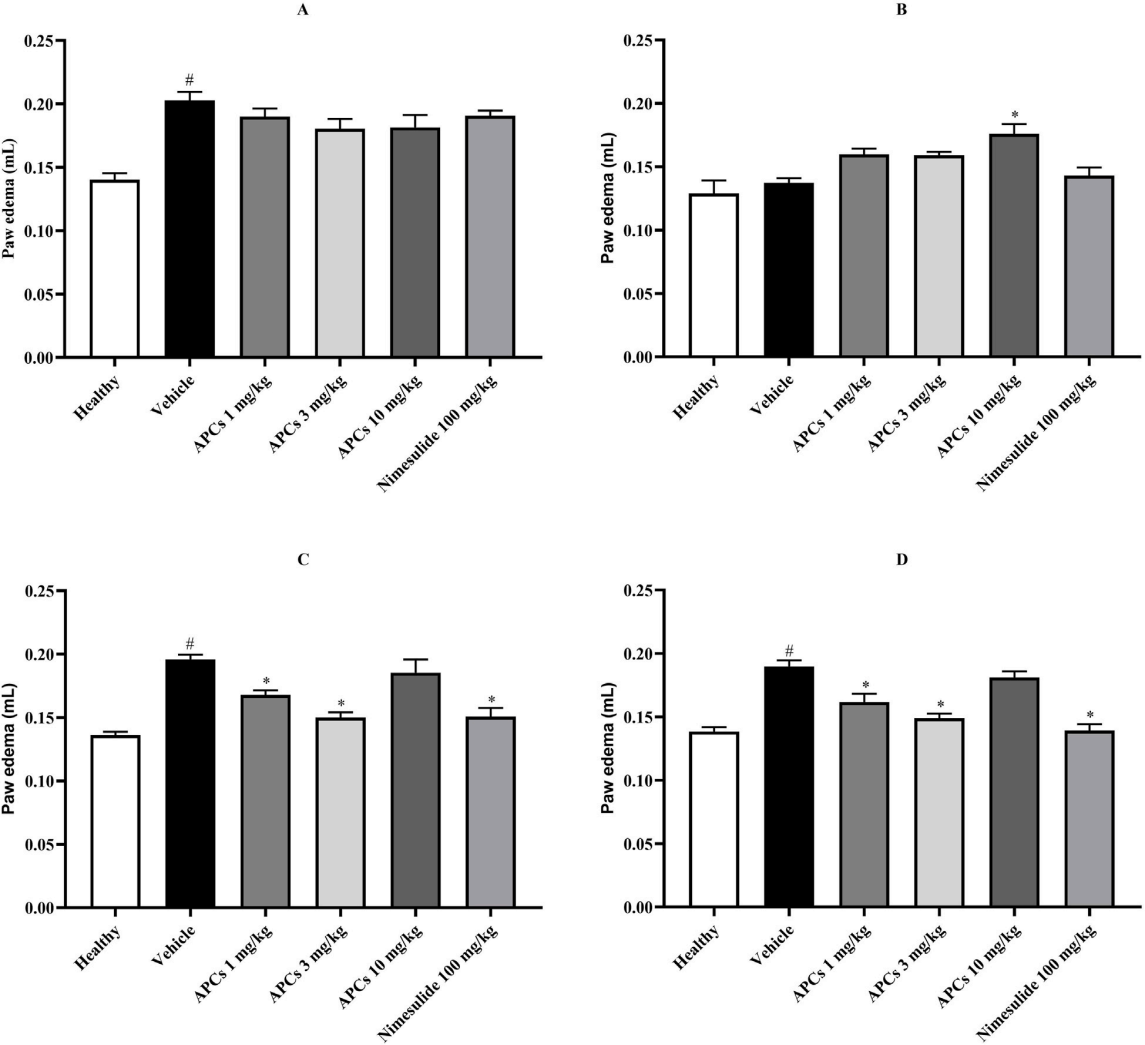
The volume of the animals’ paws after carrageenan application. Legend: Effect of treatment with APCs 1, 3, and 10 mg/kg, vehicle 10 mL/kg, and numesulide 100 mg/kg on the paw edema of animals. **(A)** The edema was analyzed 30 min after carrageenan application to the paw. **(B)** Edema was analyzed 90 min after carrageenan application to the paw. **(C)** Edema was analyzed 150 min after carrageenan application to the paw. **(D)** Edema was analyzed 210 min after carrageenan application to the paw. The results are expressed as the mean ± standard error of the mean (n = 7). *p < 0.05 compared to the negative control group by one-way ANOVA followed by Dunnett’s test. #p < 0.05 comparing the basal and negative control groups by one-way ANOVA followed by Dunnett’s test.

Carrageenan-induced inflammation in the paw of the mice was assessed based on the edema (mL) at 30, 90, 150, and 210 min (Figures 7A–D). It is important to note that the animals had similar paw volumes before carrageenan application; however, with the induction of edema, all groups that received carrageenan.

After 30 min of carrageenan application, the animals that received the vehicle had a paw volume of 0.20 ± 0.007 mL, representing a significant difference (p < 0.05) compared to the animals that received saline in the paw with a volume of 0.14 ± 0.005 mL (Figure 7A).

After 90 min of carrageenan application, the volume of the animal’s paws decreased, but the animals treated with APCs 10 mg/kg had a volume of 0.18 ± 0.008 mL, which represented a significant difference (p < 0.05), compared to the animals that received the vehicle with 0.14 ± 0.004 mL (Figure 7B).

At 150 min, the edema profile had changed, represented by a volume of 0.196 ± 0.003 mL in the animals that received vehicle and 0.168 ± 0.004, 0.150 ± 0.004, and 0.151 ± 0.007 mL for the animals treated with APCs 1 and 3 mg/kg and nimesulide 100 mg/kg, respectively, showing a significant difference (p < 0.05) between these groups compared to the animals that received vehicle (Figure 7C).

The effect profile remained the same, with the paw edema of the animals receiving vehicle being 0.19 ± 0.005 mL and the animals treated with APCs 1 and 3 mg/kg and nimesulide 100 mg/kg being 0.162 ± 0.007; 0.149 ± 0.003; and 0.139 ± 0.005 mL, respectively, which represents a significant difference (p < 0.05) between the groups (Figure 7D).

### 3.7 Evaluation of the antipyretic activity of APCs in the *S. Cerevisiae*-induced fever model

**FIGURE 8:**
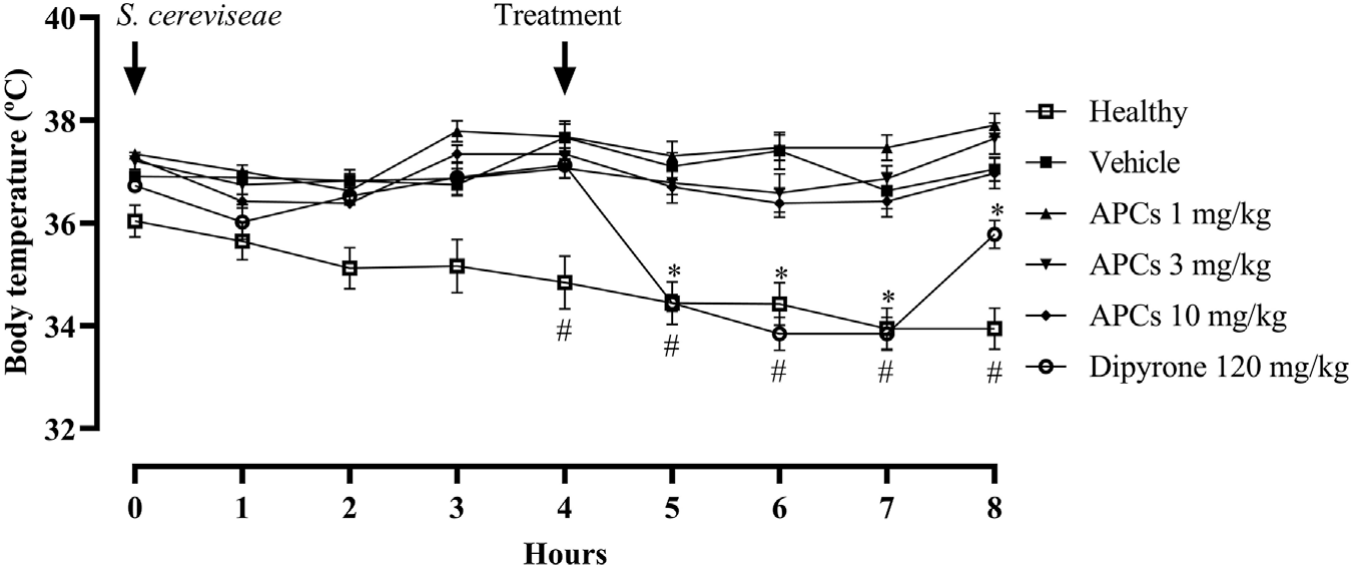
Temperature of animals after *S. cerevisiae* administration. Legend: Effect of treatment with APCs 1, 3, and 10 mg/kg, vehicle 10 mL/kg, and dipyrone 120 mg/kg on the temperature of animals administered a suspension of *S. cerevisiae*. The results are expressed as the mean ± standard error of the mean (n = 7). *p < 0.05 compared to the negative control group using two-way ANOVA followed by Dunnett’s test. #p < 0.05 comparing the baseline group with the negative control group by two-way ANOVA followed by Dunnett’s test.

In the evaluation of body temperature after the administration of a suspension containing *S. cerevisiae*, treatment with APCs at 1, 3, or 10 mg/kg was not effective in controlling yeast-induced fever (Figure 8).

The basal group (animals that received saline instead of *S. cerevisiae*) and the animals that received the vehicle, at 4 h after receiving the yeast, showed statistically significant differences, with temperatures of 34.84 °C ± 0.51 °C and 37.66 °C ± 0.26 °C, respectively.

After treatment, the animals with fever treated with APCs at doses of 1, 3, and 10 mg/kg showed no effect in the subsequent 4 h. The animals treated with dipyrone at a dose of 120 mg/kg had temperatures of 34.44 ± 0.16, 33.84 ± 0.32, 33.84 ± 0.32, and 35.78 °C ± 0.27 °C, respectively, 4 h after treatment, with a difference to the animals that received vehicle with temperatures of 37.10 ± 0.26, 37.40 ± 0.36, 36.62 ± 0.35, and 37.04 °C ± 0.23 °C, respectively, in all cases showing a significant difference (p < 0.05).

The numerical data after the febrile peak in the fourth hour until the end of the experiment are shown in Table 3.

**TABLE 3:** The temperature of the animals was measured 4 h after fever induction with *S. cerevisiae*.

Groups/Temperature	Period				
	4 h	5 h	6 h	7 h	8 h
Healthy	34.84 ± 0.510	34.44 ± 0.413	34.42 ± 0.413	33.94 ± 0.402	33.94 ± 0.402
Vehicle	37.66 ± 0.266 #	37.10 ± 0.265 #	37.40 ± 0.358 #	36.62 ± 0.351 #	37.04 ± 0.229 #
APCs 1 mg/kg	37.68 ± 0.304	37.30 ± 0.283	37.46 ± 0.246	37.46 ± 0.246	37.90 ± 0.230
APCs 3 mg/kg	37.06 ± 0.186	36.78 ± 0.233	36.58 ± 0.368	36.86 ± 0.254	37.64 ± 0.304
APCs 10 mg/kg	37.34 ± 0.117	36.70 ± 0.311	36.38 ± 0.275	36.42 ± 0.302	36.96 ± 0.301
Dipyrone 120 mg/kg	37.12 ± 0.254	34.44 ± 0.160*	33.84 ± 0.323*	33.84 ± 0.323*	35.78 ± 0.271*

Legend: *p < 0.05 when compared to the negative control group by one-way ANOVA, followed by Dunnett’s test. #p < 0.05 when comparing the baseline and the negative control groups using one-way ANOVA, followed by Dunnett’s test.

### 3.8 Antidysmenorrheal effect of APCs in a model of primary dysmenorrhea

**FIGURE 9:**
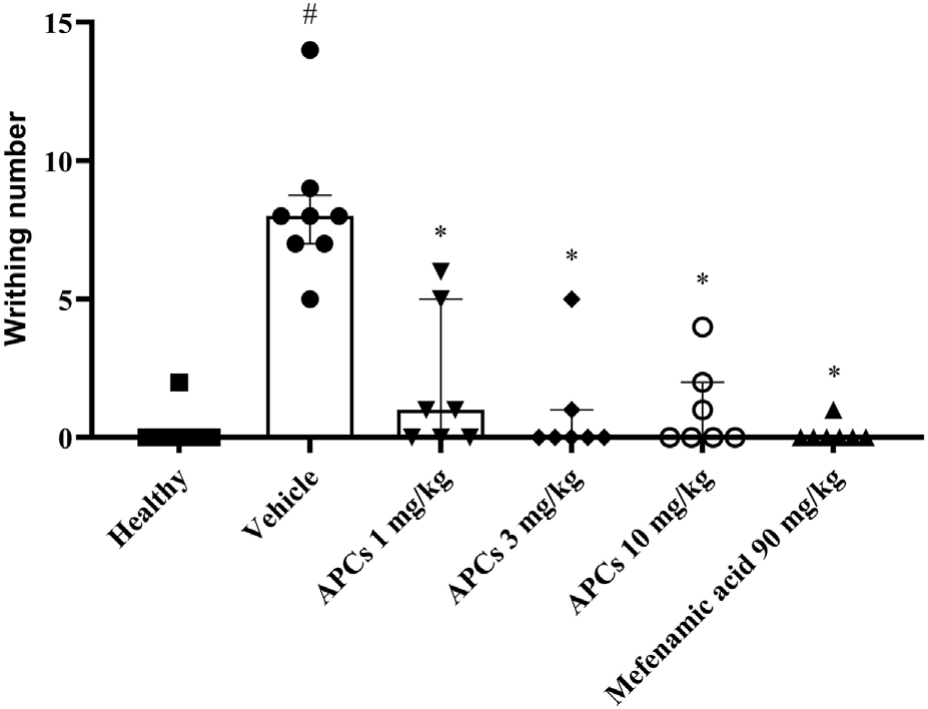
Abdominal twitching in the primary dysmenorrhea protocol. Legend: Effect of treatment with APCs 1, 3, and 10 mg/kg, vehicle 10 mL/kg, and mefenamic acid (90 mg/kg) on the abdominal twitch count in mice after receiving oxytocin 0.4 IU. Results are expressed as median with interquartile range (Q25 - Q75), n = 6. *p < 0.05 when comparing the test groups to the group that received the vehicle. Kruskal-Wallis test was performed, followed by Dunn’s post-test or Mann-Whitney test when necessary.

Regarding the anti-dysmenorrheic effect of APCs, a significant difference (p < 0.05) in abdominal contractions was observed at 3 and 10 mg/kg doses, and this effect was also observed in the animals treated with mefenamic acid 90 mg/kg (Figure 9).

The group of animals that did not receive estradiol and received only saline was considered the basal group and had a median of 0 (Q25 = 0; Q75 = 1.0) twitches, while the group that received estradiol and was treated with the vehicle had a median of 7.5 (Q25 = 5.0; Q75 = 9.5) twitches, showing a significant difference. The animals administered APCs 1 mg/kg had a median of 1.0 (Q25 = 0; Q75 = 5.0) twitches, with no significant difference. The animals given APCs 3 and 10 mg/kg had medians of 0 (Q25 = 0; Q75 = 1.0) and 0 (Q25 = 0; Q75 = 2.0), respectively, showing a significant difference (p < 0.05) compared to the group administered the vehicle. Animals treated with mefenamic acid (90 mg/kg) showed a median of 0 (Q25 = 0; Q75 = 0) abdominal contortions.

## 4 Discussion

In the literature, approximately 177 phytocannabinoids have been described in cannabis using various methods, such as LC, MS, gas chromatography (GC), nuclear magnetic resonance (NMR), or hyphenated apparatus (GC-MS, LC-MS, etc.), mainly identifying the classic cannabinoids such as CBG, CBC, CBN, CBD, THC, and their acid forms (Capriotti et al., 2021; Duchateau et al., 2025).

In addition, vitexin and other flavonoids are compounds found in high concentrations in the species, with more than 34 such compounds described in *C. sativa* (Radwan et al., 2021). CBS and other alkaloids, such as spermidine-type alkaloids have also been described in the species (Dalli et al., 2023), including in its roots (Lima et al., 2021; Araújo et al., 2024).

In behavioral studies using the OFT, it is important to mention that APCs modified the exploratory mobility behavior of mice at doses of 30 or 100 mg/kg. Maayah et al. (2022) used a *Cannabis* extract at a dose of 15 mg/kg for CBD or CBD and THC mixed, showing that the animals that received the extract with the two classic cannabinoids had a reduction in the distance traveled (Maayah et al., 2022).

The analysis of the animals’ body temperature was essential for choosing the doses in the subsequent pharmacological studies, based on Bilbrey et al. (2022), who indicated that cannabinoid activity on CB1 receptors can lead to potential behavioral changes, as well as the ability to produce acute antinociception, hypolocomotion, and hypothermia (Bilbrey et al., 2022).

This is explained by the activation of cannabinoid receptors, especially hypothalamic receptors, in the induction of hypothermia, as shown by other studies involving the administration of arachdonoylethanolamide (anandamide), cannabis extract, and Δ^9^-THC, with the effect of the latter reversed by SR141716 (rimonabant), an antagonist/inverse agonist of CB1 receptors (Borgen et al., 1973; Crawley et al., 1993; Malone and Taylor, 1998; Suzuki et al., 2012). Despite this, studies have shown that this hypothermic effect depends on the environmental temperature (Fennessy and Taylor, 1977), and others have indicated that the induction of hypothermia depends on CB1 receptors, but its maintenance is through the modulation of other pathways (Nguyen et al., 2020).

With regard to antinociceptive data, studies on *Cannabis* extracts can be found in the literature for some experimental models. In the model of abdominal contortions induced by acetic acid, Sofia, Vassar, and Knobloch (1975) showed that a crude extract of *Cannabis* had an effective dose for 50% of the animals (ED_50_) of 8.8 mg/kg (po), and that from 6.25 mg/kg, it already had an effect on reducing abdominal contortions (Sofia et al., 1975). More recently, Harris et al. (2019) prepared a *Cannabis* extract in the same experimental model and identified that doses of 4.8 and 14.5 mg/kg, administered intraperitoneally, reduced the nociceptive behavior reflected by abdominal contortions (Harris et al., 2019).

Studies involving the hot-plate test are diverse, with THC having an ED_50_ of 5 mg/kg (confidence interval 2.9–8.8 mg/kg), and in the same study, the *Cannabis* extract (Pakistani hashish with 22% THC, 43% CBD, and 34% CBN) showed ED_50_ values of 47 mg/kg (confidence interval 30.9–71.4 mg/kg) (Chesher et al., 1973). Similarly, Sofia, Vassar, and Knobloch (1975) showed that the ED_50_ of THC was 7.7 mg/kg (confidence interval 4.9–12.9 mg/kg) and that of the ethanolic extract of *Cannabis* was 8.8 mg/kg (confidence interval 4.1–18.9 mg/kg), with CBN and CBD showing values greater than 400 mg/kg (Sofia et al., 1975). Devsi et al. (2020) observed that some *C. sativa* chemotypes had a maximum effect in the hot-plate test at a dose of 3 mg/kg (Devsi et al., 2020).

Studies related to the extract of the aerial parts of *C. sativa* in the formalin protocol are scarce or non-existent; therefore, approximate data can be observed with the use of CBC 20 mg/kg (ip), in which the use of this cannabinoid reduced the area under the curve (AUC) of pain behavior in the first and second phases of the method (Raup-Konsavage et al., 2023). In a study by Blanton et al. (2022), another cannabinoid, CBD, showed an ED_50_ of 2 mg/kg (ip) in the inflammatory phase (Blanton et al., 2022). In contrast, Sepulveda et al. (2022) reported that 10 mg/kg (ip) CBD and CBG alone or together in oil were not effective in both phases of the formalin test (Sepulveda et al., 2022). These data suggest that cannabinoids isolated from *C. sativa* have antinociceptive potential, but there is an overlap when it comes to the extract since the doses are low and manage to control the pain behavior expressed by the mice.

In studies on inflammation, such as carrageenan-induced paw edema, the literature shows that the ethanolic extract of Lebanese *C. sativa* flowers at doses of 25 and 50 mg/kg reduced edema (Shebaby et al., 2021). In studies with THC 1 mg/kg (po), a reduction in the size of paw edema in rats was observed and a synergistic effect was observed when the ineffective doses of CBDA 0.1 mg/kg and THC 0.1 mg/kg were administered simultaneously and promoted a reduction in edema (Rock et al., 2018).

In a study conducted by our group using the ethanolic extract of *C. sativa*, hypothermic effect was observed; however, when non-hypothermic doses were used in the brewer’s yeast-induced fever method, there was no antipyretic effect. However, it is possible to observe in the study carried out with THC by Kosersky et al. (1973), that at doses of 6.3, 12.5, 25, and 50 mg/kg the administration of this cannabinoid led to a reduction in the body temperature of rats (Kosersky et al., 1973). This hypothermic effect is normally associated with the activation of CB1 receptors in the preoptic center of the hypothalamus in rats, mice, and primates (Rawls et al., 2002).

Concluding the studies on inflammation, in this case, uterine inflammation for the primary dysmenorrhea, demonstrate the importance of the endocannabinoid system for the menstrual cycle and uterine contractility. Pagano et al. (2017) demonstrated the difference in endocannabinoid mediators depending on the phase of the menstrual cycle in female mice, as well as the influence of cannabinoid receptors on the inhibition of uterine contraction, which is more influenced by the stimulation of CB1 receptors and, to a lesser extent, CB2 receptors (Pagano et al., 2017).

Furthermore, in a review conducted by Seifalian et al. (2022), the researchers raised the problem of dysmenorrhea and the use of cannabis as a therapeutic alternative. In this study, the researchers supported the large-scale use of CBD and THC, isolated in a formulation with two cannabinoids (Seifalian et al., 2022).

The only experimental study on the use of *C. sativa* in an experimental model of dysmenorrhea was conducted by Araújo et al. (2024), who used an aqueous extract of the plant’s roots and showed a promising pharmacological effect, indicating the potential of the whole plant in this treatment (Araújo et al., 2024).

This study presents a considerable amount of data on the peripheral effects of APCs; however, there are important limitations, such as the impossibility of investigating the mechanism of action of the extract, confirmation by chemical studies of more compounds, the lack of standardization of plants obtained naturally from illegal plantations in Brazil, and the investigation of other compounds present in the plant, soil, or water, such as fertilizers, pesticides, or chemical contaminants.

## 5 Conclusion

Our results suggest that APCs contain classic cannabinoids, flavonoids, and alkaloids, and that classic cannabinoids, THC, and CBD are present. The administration of APCs promoted behavioral changes in the animals consistent with the pharmacological effects of these substances, such as reduced ambulation and hypothermic effect at doses of 30 and 100 mg/kg. In pharmacological studies, antinociceptive, anti-inflammatory, and anti-dysmenorrheal effects were observed in different experimental models and in the 1–10 mg/kg dose range; however, the APCs failed to show an antipyretic effect at these doses.

This study demonstrated the pharmacological effects of APCs administered in doses that are suggested not to induce events in the central nervous system, and the mechanisms by which these events occur should be further investigated.

New studies are important for investigating the mechanism of action of APCs using pharmacological blockers to observe whether the effects are directly related to cannabinoid receptors or other biochemical targets that directly influence experimental models. In addition, the data show an intrinsic correlation with the non-psychoactive medicinal use of *C. sativa*, which may benefit individuals predisposed to the plant’s intense effects on the central nervous system.

## Data Availability

The original contributions presented in the study are included in the article/supplementary material, further inquiries can be directed to the corresponding author.
